# Secretory form of viral protease NIa ameliorates amyloid-β pathology and cognitive deficits in a mouse model of Alzheimer’s disease

**DOI:** 10.3389/fnagi.2026.1720518

**Published:** 2026-04-22

**Authors:** Euy Jun Park, Bo-Ram Mun, Sung Yoon Kim, Muthukumar Elangovan, Sung Bin Kim, Won-Seok Choi, Woo Jin Park

**Affiliations:** 1 School of Life Sciences, Gwangju Institute of Science and Technology, Gwangju, Republic of Korea; 2 School of Biological Sciences and Technology, Chonnam National University, Gwangju, Republic of Korea

**Keywords:** 5xFAD mice, Alzheimer’s disease, amyloid-β, amyloid-β degrading protease, nuclear inclusion a

## Abstract

Alzheimer’s disease (AD), the leading cause of dementia, is characterized by extracellular amyloid-β (Aβ) accumulation. Immunotherapies targeting Aβ clearance show promise, highlighting the therapeutic value of enhancing Aβ removal. We previously identified that nuclear inclusion a (NIa), a plant viral protease, fortuitously cleaves Aβ with strict sequence specificity. Here, we engineered a secretory form, SecNIa, to degrade extracellular Aβ. SecNIa was efficiently secreted from transfected cells while retaining potent Aβ-cleaving activity. Adeno-associated virus (AAV)-mediated delivery of SecNIa into 5xFAD mice resulted in robust hippocampal expression and cerebrospinal fluid secretion. SecNIa expression significantly reduced soluble and insoluble Aβ, decreased hippocampal plaques, and improved cognition, fully normalizing recognition memory and enhancing spatial learning. These findings establish SecNIa as a promising therapeutic strategy to directly target pathogenic extracellular Aβ in AD.

## 1 Introduction

Alzheimer’s disease (AD) is a progressive neurodegenerative disorder that affects approximately 55 million people worldwide, making it the leading cause of dementia (Alzheimer’s Disease International, 2024). Clinically, AD is characterized by progressive memory loss and cognitive decline, while its pathological hallmarks include extracellular amyloid plaques, intracellular neurofibrillary tangles composed of hyperphosphorylated tau, and neuroinflammation (Querfurth and LaFerla, 2010). The amyloid cascade hypothesis proposes that excessive amyloid-β (Aβ) deposition initiates the pathological cascade that ultimately leads to symptomatic dementia (Hardy and Higgins, 1992). Indeed, Aβ accumulation precedes other hallmarks of AD in clinical progression (Jack et al., 2010; Koscik et al., 2020).

Recent FDA approvals of Aβ-targeting immunotherapies such as lecanemab (Leqembi) and donanemab (Kisunla) (van Dyck et al., 2023; Sims et al., 2023)—which reduce amyloid burden and modestly slow cognitive decline— have validated Aβ as a mechanistically relevant and modifiable target in the pathogenesis of AD. However, the limited efficacy and safety concerns associated with these antibodies (e.g., amyloid-related imaging abnormalities, ARIA) underscore the need for complementary and safer therapeutic approaches.

Aβ exists in multiple forms, including monomers, oligomers, fibrils, and plaques. Although amyloid plaques are a defining feature of AD pathology, oligomeric Aβ is considered the most toxic species (Cline et al., 2018). For example, oligomeric Aβ was shown to be 10- and 40-fold more cytotoxic to neuronal cells than fibrillar and monomeric Aβ, respectively (Lambert et al., 1998). Similarly, transgenic mice carrying the Osaka mutation (APP E693Δ), selectively increases oligomer levels, exhibit abnormal tau phosphorylation, gliosis, and neuronal loss (Tomiyama et al., 2008).

Given the success of immunotherapies, enhancing enzymatic degradation of Aβ species also represents a promising, though relatively underexplored therapeutic avenue. Age-related decline in Aβ clearance, including reduced expression of Aβ-degrading enzymes, is thought to be a major contributor to amyloid accumulation in AD (Sehar et al., 2022; Miners et al., 2008; Glabe, 2000). Several endogenous proteases regulate Aβ homeostasis in the brain (Saido and Leissring, 2012), with neprilysin (NEP) acting as a key physiological regulator. In mice, NEP inhibition elevates Aβ levels and induces hippocampus-dependent memory deficits and AD-like pathology (Iwata et al., 2001), whereas viral-mediated NEP overexpression reduces plaque burden and improves cognition (Iwata et al., 2013). However, results have been mixed: in some models, NEP overexpression failed to reduce oligomeric Aβ or prevent cognitive decline (Meilandt et al., 2009), and NEP knockout paradoxically improved memory performance due to accumulation of other physiological peptides normally degraded by NEP (Walther et al., 2009). Thus, an enzyme capable of selectively cleaving toxic Aβ oligomers with high substrate specificity represents a unique therapeutic opportunity.

Nuclear inclusion a (NIa) is a cytosolic protease of turnip mosaic virus (TuMV) from the *Potyviridae* family, where it processes the viral polyprotein during maturation (Riechmann et al., 1992). We previously reported that NIa exhibits strict substrate specificity for the Val-Xaa-His-Gln consensus sequence (Kang et al., 2001). Intriguingly, further studies revealed that NIa fortuitously cleaves the Val-His-His-Gln motif within Aβ, efficiently degrading both the monomeric and oligomeric forms *in vitro* (Han et al., 2010). This serendipitous discovery further led to findings that NIa expression prevents Aβ oligomer-induced cytotoxicity *in vitro* (Shin et al., 2014) and reduces Aβ burden and behavioral deficits in APP/PS1 mice (Kim et al., 2012).

In the present study, we extend this work by engineering NIa as a secreted fusion protein, termed secretory NIa (SecNIa), to enable direct proteolytic activity against extracellular Aβ species. We demonstrate that SecNIa expression reduces Aβ burden and ameliorates cognitive deficits in AD model mice, providing a novel therapeutic opportunity for the treatment of AD.

## Materials and methods

### 2.1 Plasmid construction

All plasmid synthesis was performed at Cosmogenetech (South Korea). The GOI sequences of NIa and SecNIa were subcloned into pcDNA4/myc-His for downstream detection by Myc-tag and protein purification by 6xHis-tag. SecNIa is a fusion protein consisting of an Igκ leader sequence, a human IgG1 Fc fragment, and the TuMV NIa proteolytic domain (a.a. 9–222). Mutations in the human Fc fragment that minimize Fc gamma receptor (FcγR) binding and complement activation (E233P, L234V, L235A, G236Δ, A327G, A330S, P331S) were adopted for immunological inertia, as previously reported (Armour et al., 1999; Armour et al., 2003). For the fluorogenic peptide cleavage studies, an additional construct with the Igκ leader sequence at the N-term of NIa was synthesized in order to facilitate secretion and allow purification of the protein.

### 2.2 Cell culture and transfection

AD293 cells—a derivative of the HEK293 cell line with improved cell adherence—were maintained in high glucose Dulbecco’s Modified Eagle Medium (DMEM) supplemented with 10% fetal bovine serum (FBS) and 1% antibiotic-antimycotic solution (100X, Gibco, United States) at 37°C in a humidified atmosphere with 5% CO_2_. Cells were seeded a day before transfection, and 24 h later, plasmids expressing NIa or SecNIa were transfected into AD293 cells using Lipofectamine 2000 (Thermo Scientific, United States) according to the manufacturer’s instructions. Culture media were collected 24 h post-transfection, and cells were washed and stored in −80°C until further preparation.

A derivative of HEK293 cells adapted to suspension culture (HEK293F) were maintained in unsupplemented Freestyle 293 media (Gibco, United States) at 37°C in a shaking incubator with 8% CO_2_. Cells were seeded a day before transfection, and 24 h later, cells were transfected with plasmids expressing NIa and SecNIa using FectoPro (Polyplus, France) according to the manufacturer’s instructions. 3 days following transfection, the culture media was isolated for protein purification.

### 2.3 Electrophoresis and western blotting

Cell lysates were harvested and lysed by sonication in RIPA buffer (150 mM NaCl, 10 mM NaF, 1% NP-40, 0.5% sodium deoxycholate, 0.1% SDS, 50 mM Tris, pH 7.4) containing 1X protease inhibitor cocktail (Sigma, United States). For SDS-PAGE, all sample protein concentrations were measured by BCA quantification and separated using 4–12% gradient gels (Invitrogen, United States). Following transfer, membranes were blocked in 5% skim milk solution and incubated with antibodies against Myc-tag (2272 and 2276S, CST, United States) and GAPDH (2118, CST, United States).

### 2.4 Purification of recombinant NIa and SecNIa protein

The culture media obtained from transfected HEK293F cells were incubated overnight at 4°C with Ni-NTA agarose (Qiagen, Germany) to a bead-media ratio of 1:200 to capture the His-tagged NIa and SecNIa protein. The beads were washed four times with wash buffer (20 mM HEPES, 200 mM NaCl, 1 mM EDTA, pH 7.9) and eluted using 500 mM imidazole. The protein was concentrated and imidazole removed by using a Pierce™ protein concentrator (88517, Thermo Scientific, United States) in conjunction with buffer exchange.

### 2.5 Fluorogenic peptide cleavage assay

A FRET-based peptide mimicking the Aβ_10–19_ sequence (YEVHHQKLVF) was synthesized through AnaSpec (United States) by using an EDANS fluorophore and Dabcyl quencher, as described previously (Zou et al., 2005). The cleavage activity of NIa and SecNIa were assessed by incubating various concentrations of purified protein with 30 μM of the FRET peptide in an assay buffer tailored to NIa protease (20 mM HEPES, 10 mM KCl, 10 mM MgCl_2_, 1 mM DTT, 0.2 mg/mL BSA, pH 8.3 at 12°C) (Kim et al., 1996). Fluorescence of the fluorophore was monitored over time at 340 nm excitation and 505 nm emission using a spectrophotometer, with the increase in fluorescence intensity indicating peptide cleavage.

### 2.6 AAV production

AAV9 vectors containing Myc-tagged SecNIa or full-length human NEP gene driven by a CMV promoter and incorporating a Woodchuck Hepatitis Virus post-transcriptional regulatory element (WPRE) for enhanced gene expression was generated through Virovek (United States). For negative controls, AAV9 virus-like particles (VLP) and AAV9 expressing GFP under the control of the CMV promoter were also purchased from Virovek.

### 2.7 Animals

5xFAD transgenic AD model mice overexpressing human mutated APP and PS1, as previously described (Oakley et al., 2006), were maintained in the B6/SJL genetic background. Animals were kept in temperature and humidity-controlled housing with a 12-h light/dark cycle, and had *ad libitum* access to food and water. For all experiments, only male 5xFAD mice were utilized. To examine the effects of SecNIa on Aβ burden, 5xFAD mice were randomized into 5xFAD/VLP (*n* = 4), and 5xFAD/SecNIa (*n* = 4), along with wild-type (WT) littermates (*n* = 4). Mice were administered respective AAV at 2 months of age, and sacrificed at 2 months post-injection for downstream biochemical analysis. To examine the effects of SecNIa on cognitive deficits, 5xFAD mice were randomized into 5xFAD/GFP (*n* = 8), and 5xFAD/SecNIa (*n* = 6), along with WT littermates (*n* = 7) prior to respective AAV administration at 2 months of age, and behavioral tests starting at 6 months post-injection. All animal experiments adhered to institutional guidelines and received approval from the ethics committee at Gwangju Institute of Science and Technology (GIST).

### 2.8 Stereotaxic injection

Stereotaxic injection into the lateral ventricles of the mouse brain were performed as previously described (Kim et al., 2024). Briefly, a mixture of 95 mg/kg ketamine and 5 mg/kg xylazine was used to anesthetize mice via intraperitoneal (i.p.) injection. After being secured to a stereotax, a total of 1 × 10^11^ viral genomes (vg) of the respective AAV vectors in a 10 μL volume, were infused bilaterally into the lateral ventricles (coordinates from Bregma in mm: AP, −0.46; ML, ± 1.00; DV, −2.20).

### 2.9 Collection of mouse CSF

Cerebrospinal fluid (CSF) was collected from the cisterna magna of anesthetized mice prior to transcardial perfusion as previously described (Lim et al., 2018). Briefly, mice were positioned in a stereotaxic frame with the head sharply flexed. After a small incision was made to expose the cisterna magna membrane, CSF was carefully aspirated using a pulled glass capillary tube. Samples contaminated with blood were visually recorded and removed from analysis. Collected CSF samples were immediately flash-frozen in liquid nitrogen and stored at −80°C until analysis.

### 2.10 Tissue preparation

Harvest and tissue preparation of mice brains were carried out as previously described (Kim et al., 2024). Mice were anesthetized by avertin (250 mg/kg) via i.p. injection and confirmed for deep anesthesia, then euthanized by transcardial perfusion with PBS. Following extraction of the brain and dissection along the midsagittal plane, the hippocampus of the left hemisphere was isolated and snap-frozen in liquid nitrogen, while the right hemisphere was fixed with 4% paraformaldehyde at 4°C for 24 h. For biochemistry, hippocampal lysates were prepared by homogenization and sonication of individual hippocampi in RIPA buffer with 2X protease inhibitor cocktail (Sigma, United States), and centrifuged to remove debris. For histological analysis, the post-fixed right hemisphere was washed with PBS, cryoprotected in 30% sucrose, and embedded in OCT (Leica, Germany). 20 μm-thick coronal sections were made with a cryostat (HM525 NX, Thermo Scientific, United States). Lysates and tissue sections were stored in −80°C until further analysis.

### 2.11 Immunohistochemistry and image analysis

Antigen retrieval was performed on the sections using a citrate buffer (10 mM trisodium citrate, 0.05% Tween 20, pH 6.0), followed by blocking and permeabilization using a 5% goat serum, 5% BSA, 0.15% Triton X-100 solution in PBS at RT for 1 h. Sections were then incubated with anti-Myc (1:1,000) (2722, CST, United States) and/or anti-Aβ (N-term) (1:500) (18584, IBL, United States) antibodies at 4°C for 48 h. Finally, the sections were incubated in Alexa 488-conjugated anti-mouse IgG (1:1,000) (A11001, Invitrogen, United States) and/or Alexa 555-conjugated anti-rabbit IgG (1:1,000) (A21428, Invitrogen, United States) antibodies along with Hoechst 33342 at RT for 1.5 h before mounting and microscopy. All image analyses following IHC were performed through ImageJ software (NIH) as previously described (Kim et al., 2024).

### 2.12 Assessment of Aβ levels

Aβ_1–40_ and Aβ_1–42_ concentrations were measured using Human Amyloid-β (1–40) (FL) Assay Kit (27718, IBL, Japan) and Human Amyloid-β (1–42) (FL) Assay Kit (27719, IBL, Japan), respectively. Procedures and measurements were performed according to the supplier instructions.

### 2.13 Behavior studies

Behavior tests were performed as described previously (Kim et al., 2024). The mice injected with AAV vectors at 2 months of age were subjected to behavior tests at 8 months of age. All video analysis of mice behavior was performed using ANY-maze software (Stoelting, United States).

#### 2.13.1 Open field test

To measure locomotor activity, the open field test was performed in an open-top opaque box. Mice were placed at the center of the arena, and were allowed to freely explore the arena for 20 min with distance traveled being recorded.

#### 2.13.2 Novel object recognition test

The novel object recognition test was performed in the same open-top opaque box of the open field test. During training, two identical objects were placed at the center of the box, with mice being placed between the objects and allowed to explore for 8 min. During the test phase on the next day, one object was replaced with a novel object. Mice were again placed between the objects and were allowed to explore for 8 min while being recorded. Preference index was calculated as a percentage using time spent exploring the novel object (n) divided by the time spent exploring both familiar and novel objects (n + f).

#### 2.13.3 Morris water maze and probe test

Morris water maze test was performed using a circular water pool filled with opaque water, with two visual cues attached to the pool wall. The escape platform was placed in one quadrant 1.5 cm below the water surface. Acquisition consisted of 4 daily trials over 4 consecutive days. In each trial, mice were placed in any quadrant of the pool excluding the platform quadrant, and allowed to swim for 60 s under recording. Mice that did not reach the platform within 60 s were shifted to the platform and allowed to stay for 10 s to remember the platform location. Time taken to reach the platform (escape latency) was recorded. 24 h after the acquisition period, a probe test was performed to test for spatial memory. After removing the platform, the time mice spent in each quadrant was recorded over a period of 90 sec.

### 2.14 Statistics

Statistical analysis was performed using GraphPad Prism 10 (GraphPad Software, United States). Two-sample comparisons were made using Student’s *t*-test, while multiple comparisons were made using one-way or two-way ANOVA followed by Tukey’s *post-hoc* tests. For all analyses, statistical significance was set at *p* < 0.05.

## 3 Results

### 3.1 Design and characterization of SecNIa

**FIGURE 1:**
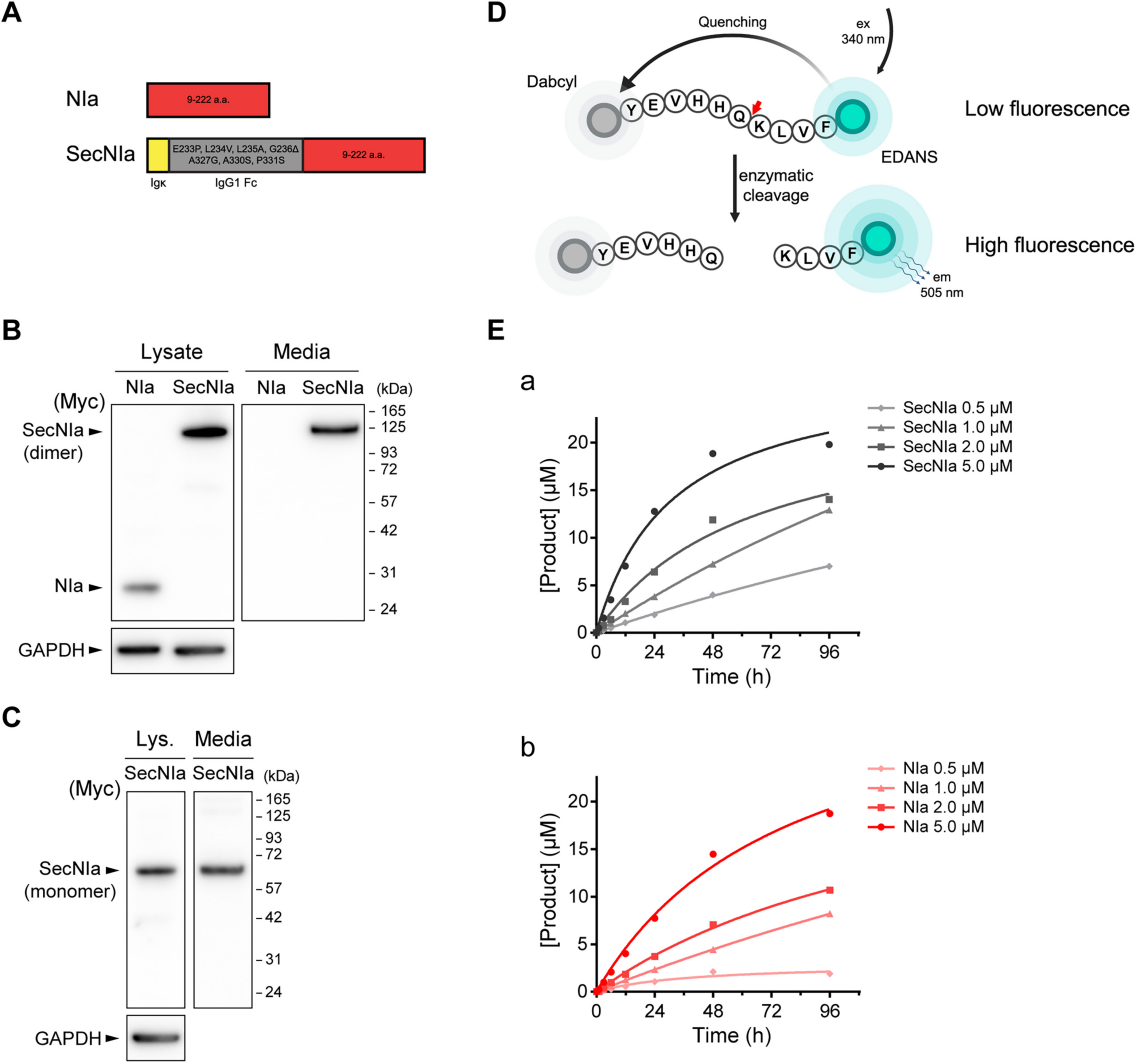
Design and characterization of SecNIa. **(A)** Schematic diagram of SecNIa. The cytoplasmic protease NIa (red) was fused to a human IgG Fc fragment (gray), and modified for secretion into the extracellular space by addition of an Igκ leader sequence (yellow). **(B)** Western blotting of cell lysates and culture media from AD293 cells transfected with plasmids encoding NIa and SecNIa under non-reducing conditions. **(C)** Western blotting of cell lysates and culture media from AD293 cells transfected with plasmid encoding SecNIa under reducing conditions. **(D)** Schematic diagram of the FRET substrate comprising the amino acid sequence of Aβ_10–19_ flanked by fluorophore (EDANS) and quencher (Dabcyl). Enzymatic cleavage of the substrate relieves quenching, resulting in increased fluorescence. Red arrow indicates the NIa cleavage site. **(E)** Time-course analysis of FRET substrate cleavage (30 μM) incubated with indicated concentrations of purified SecNIa (panel a) and NIa (panel b). The amount of cleaved substrate was plotted against time.

NIa is a cytosolic protease that can cleave Aβ with high specificity (Kang et al., 2001). In this study, we sought to engineer a secreted form of NIa capable of targeting extracellular Aβ. To accomplish this, NIa was fused with an Igκ leader sequence and a modified human IgG Fc fragment (hFc) to promote secretion and enhance stability. We refer to this engineered fusion protein as secretory NIa (SecNIa, Figure 1A).

To evaluate the expression and secretion of SecNIa, AD293 cells were transfected with plasmids encoding Myc-tagged NIa or SecNIa. Cell lysates and culture media were analyzed by non-reducing SDS-PAGE followed by western blotting. Anti-Myc antibody detected a single band of ∼27 kDa for NIa in lysates and a single band of ∼125 kDa for SecNIa in lysates and media. Notably, only SecNIa was detected in culture media, indicating that it was efficiently secreted (Figure 1B). Under reducing conditions, a single ∼65 kDa band was detected in both lysates and media of SecNIa-transfected cells, consistent with monomeric SecNIa (Figure 1C).

To determine whether SecNIa still retained sequence-specific proteolytic activity, we employed a fluorescence resonance energy transfer (FRET)-based assay (Zou et al., 2005). A FRET peptide was designed using the Aβ_10–19_ sequence (YEVHHQKLVF), which was flanked by EDANS (fluorophore) and Dabcyl (quencher) (Figure 1D). Recombinant NIa and SecNIa proteins were purified from HEK293F cells. Incubation of the FRET peptide with purified NIa or SecNIa led to a time- and concentration-dependent increase in fluorescence, confirming efficient and specific peptide cleavage. Notably, SecNIa exhibited cleavage activity comparable to native NIa, indicating that the fusion modification did not impair its proteolytic activity (Figure 1E).

Collectively, these results demonstrate that SecNIa is efficiently secreted from cells, while fully retaining Aβ-cleaving activity.

### 3.2 AAV-mediated expression of SecNIa in 5xFAD mice

**FIGURE 2:**
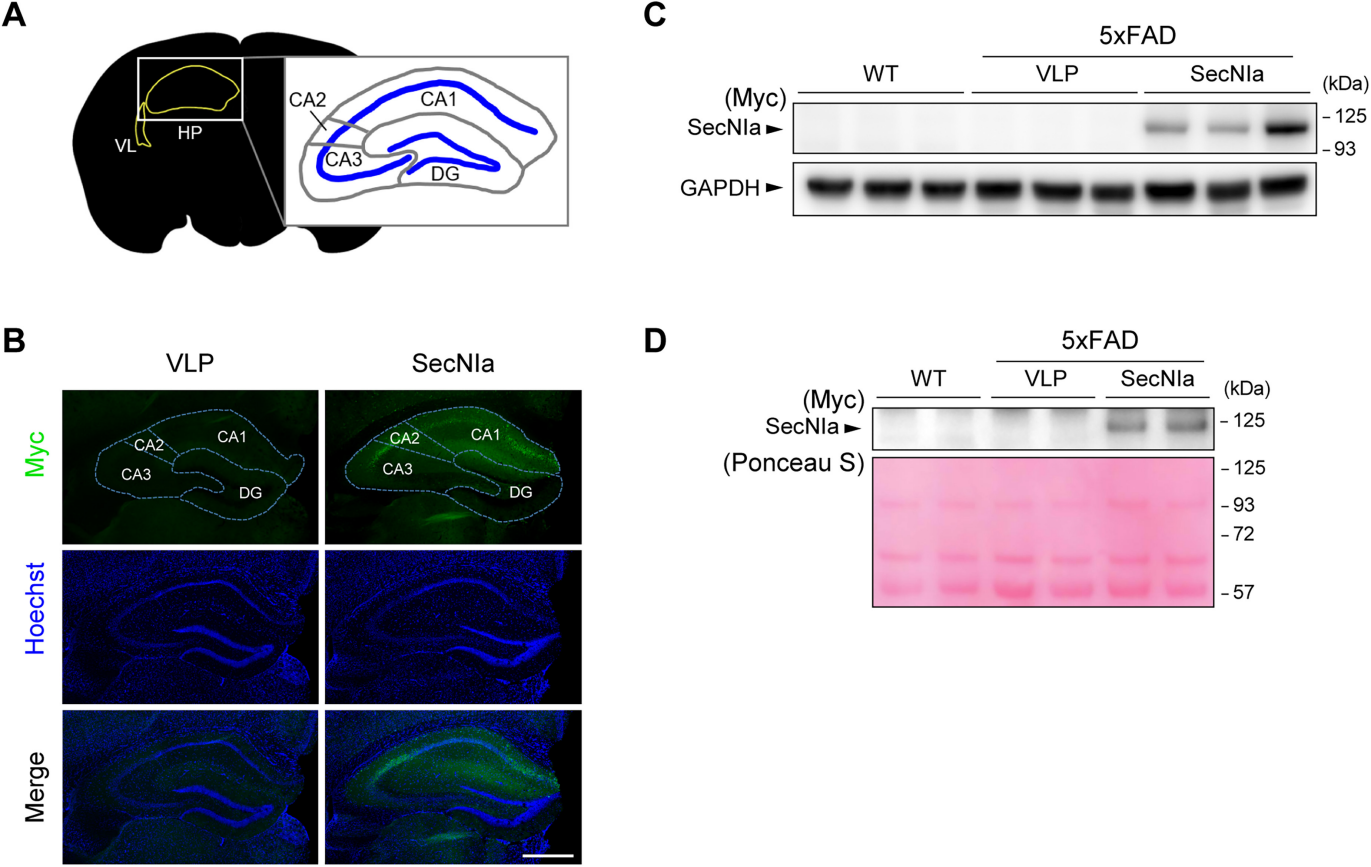
AAV-mediated expression of SecNIa in 5xFAD mice. **(A)** Simplified scheme of a coronal mouse brain section highlighting hippocampal anatomy. VL, lateral ventricle and route of AAV delivery; HP, hippocampus; CA, cornu ammonis; DG, dentate gyrus. **(B)** Representative 20 μm coronal sections of 5xFAD mouse brains 2 months after AAV-SecNIa injection, stained by IHC with anti-Myc-tag (green). SecNIa expression was detected across all CA subregions of the hippocampus, with particularly strong expression in CA1 and CA2. Scale bar, 500 μm. **(C)** Western blotting of hippocampal lysates from age-matched WT mice and 5xFAD mice injected with AAV-VLP or AAV-SecNIa under non-reducing conditions. **(D)** Western blotting of pooled CSF samples (*n* = 3) from age-matched WT mice and 5xFAD mice injected with AAV-VLP or AAV-SecNIa under non-reducing conditions. Repeated lanes indicate technical duplicates. Ponceau S staining was used as loading control.

To evaluate the therapeutic potential of SecNIa, a recombinant adeno-associated virus (AAV) vector encoding Myc-tagged SecNIa (AAV-SecNIa) under the control of the CMV promoter was generated. AAV-SecNIa was stereotaxically injected into the lateral ventricles adjacent to the hippocampus of 5xFAD mice at 2 months of age (Figure 2A). Virus-like particle (VLP) was injected in the same manner as a negative control. Two months post-injection, whole brain and hippocampus were separately harvested, and cerebrospinal fluid (CSF) was collected. Immunohistochemistry (IHC) with anti-Myc antibody revealed robust SecNIa expression within the hippocampus, particularly in the CA1/2 regions (Figure 2B). Non-reducing SDS-PAGE followed by western blotting of hippocampal lysates confirmed the efficient expression of SecNIa, with an apparent molecular weight of ∼125 kDa (Figure 2C). SecNIa was also detected in CSF, demonstrating its efficient secretion into the extracellular space of the brain (Figure 2D).

In summary, AAV-SecNIa delivery led to robust expression and secretion of SecNIa in the hippocampus of 5xFAD mice.

### 3.3 SecNIa reduces Aβ burden in 5xFAD mice

**FIGURE 3:**
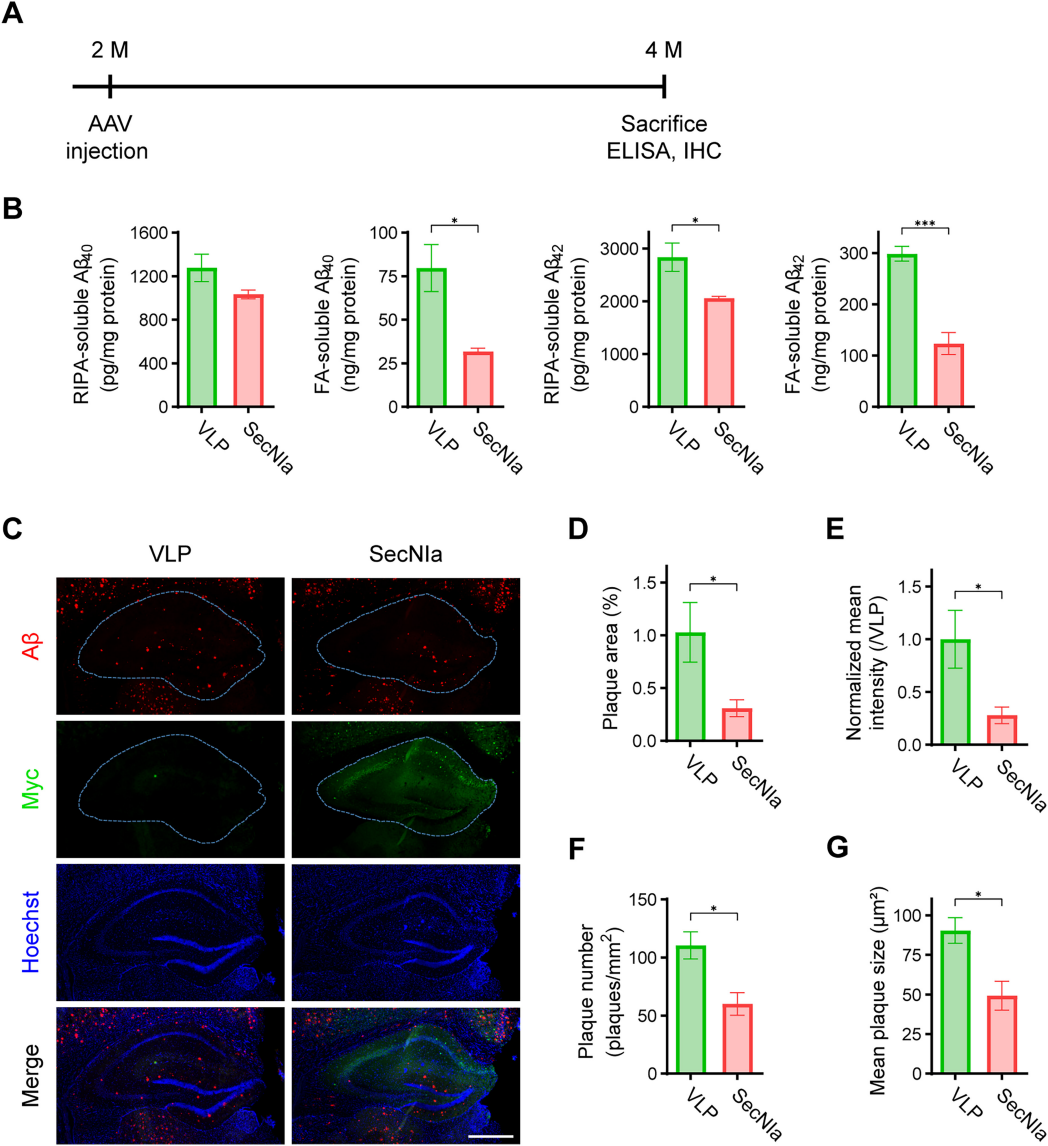
SecNIa reduces Aβ burden in 5xFAD mice. **(A)** Experimental scheme of stereotaxic AAV delivery and mouse sacrifice. 5xFAD mice were injected at 2 months of age and sacrificed 2 months later for biochemical analysis. AAV-VLP-injected 5xFAD mice (VLP) were used as a control for comparison with AAV-SecNIa-injected 5xFAD mice (SecNIa). **(B)** ELISA of Aβ_1–40_ and Aβ_1–42_ levels in RIPA and FA-soluble fractions of hippocampal lysates from VLP or SecNIa groups. **(C)** Representative 20 μm coronal mouse brain sections subjected to IHC with anti-Aβ (N-term, red) and anti-Myc-tag (green). The hippocampal region (dotted outline), showing robust SecNIa expression, displayed a marked reduction in Aβ plaques. Scale bar, 500 μm. **(D–G)** Quantitative analysis of anti-Aβ IHC images in the hippocampus: **(D)** Percentage area covered by plaques, **(E)** Mean red fluorescence intensity normalized to VLP, **(F)** Plaque count per mm^2^ tissue, and **(G)** Mean plaque size. Student’s *t*-test: *p < 0.05, ***p < 0.001. Bars and error bars represent means ± SEM. Number of animals used (all male) 5xFAD/VLP, 4; 5xFAD/SecNIa, 4.

We next examined the effect of SecNIa expression on Aβ burden. 5xFAD mice were injected with AAV-SecNIa or VLP, and sacrificed 2 months later (Figure 3A). Hippocampal lysates were fractionated into soluble (RIPA-soluble) and insoluble (FA-soluble) fractions, and Aβ levels were measured by ELISA. Aβ_1–40_ levels were reduced in both fractions of AAV-SecNIa-injected mice compared with controls, although the reduction in the RIPA-soluble fraction was not statistically significant (Figure 3B, upper panels). Aβ_1–42_ levels were significantly reduced in both fractions AAV-SecNIa-injected mice (Figure 3B, lower panels).

Consistent with these findings, IHC revealed reduced Aβ plaque deposition in AAV-SecNIa-injected mice (Figure 3C). Quantitative image analysis demonstrated significant decreases in both the Aβ plaque area and intensity within the hippocampus (Figures 3D,E). Further analysis also showed significant reductions in plaque number and mean plaque size (Figures 3F,G). Upon inspection of plaque morphology, both compact and dense plaque numbers were also significantly reduced (Supplementary Figure 1).

Taken together, these data demonstrate that AAV-SecNIa expression effectively reduces Aβ burden in 5xFAD mice.

### 3.4 SecNIa ameliorates cognitive deficits in 5xFAD mice

**FIGURE 4:**
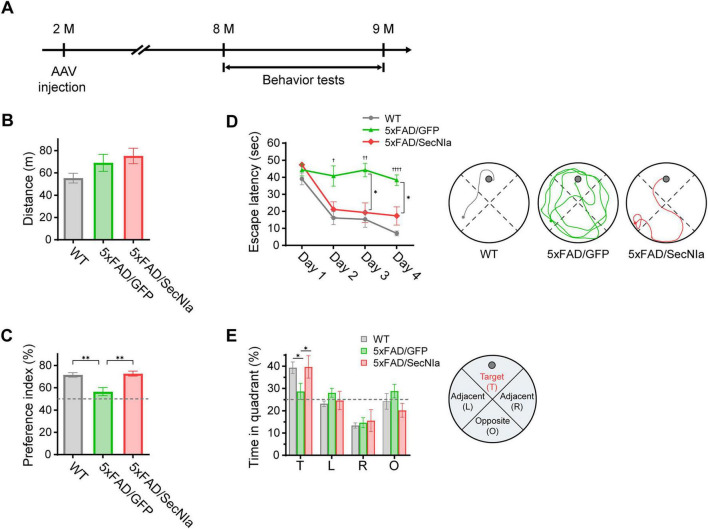
SecNIa attenuates cognitive deficits in 5xFAD mice. **(A)** Experimental scheme of stereotaxic AAV delivery and behavior tests. 5xFAD mice were injected at 2 months of age and subjected to behavior tests 6 months later. WT littermates and AAV-GFP-injected 5xFAD mice (5xFAD/GFP) were used as controls for comparison with AAV-SecNIa-injected 5xFAD mice (5xFAD/SecNIa). **(B)** Open-field test assessing locomotive activity. No significant differences in total distance traveled were observed. **(C)** Novel object recognition test evaluating recognition memory. Impaired preference for novel objects in 5xFAD/GFP mice was rescued in 5xFAD/SecNIa mice. One-way ANOVA: **p < 0.01. **(D)** Morris water maze tests of spatial learning. Escape latencies were significantly elevated in 5xFAD/GFP mice and were reduced in 5xFAD/SecNIa mice. Right panel, representative track plots of denoted groups. Two-way repeated measures ANOVA: *p < 0.05, 5xFAD/GFP vs. 5xFAD/SecNIa; ^†^ *p* < 0.05, ^††^ *p* < 0.01, ^††††^ *p* < 0.0001 WT vs. 5xFAD/GFP. **(E)** Probe test assessing spatial memory. With the platform removed, WT and 5xFAD/SecNIa mice spent significantly more time in the target quadrant compared to 5xFAD/GFP mice. Right panel, schematic of the quadrants. Two-way ANOVA: *p < 0.05. Bars or data points represent means ± SEM. Number of animals used (all male) WT, 7; 5xFAD/GFP, 8; 5xFAD/SecNIa, 6.

To determine whether reduced Aβ burden translated into cognitive improvement, 5xFAD mice were stereotaxically injected with AAV-SecNIa at 2 months of age and subjected to behavioral tests 6 months later (Figure 4A). AAV-GFP-injected 5xFAD mice and wild-type littermates (WT) were used as negative and positive controls, respectively.

Locomotor activity and anxiety-like behavior were assessed using the open-field test. No significant differences were observed in total distance traveled among the three groups (Figure 4B). Similarly, time spent in the center versus corners of the test arena did not differ among groups (data not shown). These results indicate that SecNIa expression did not adversely affect locomotive ability or anxiety levels in 5xFAD mice.

To assess recognition memory, a novel object recognition test was performed. Cognitively healthy mice typically spend more time exploring a novel object than a familiar one (Lueptow, 2017). As expected, WT mice showed a strong preference for the novel object. In contrast, AAV-GFP-injected 5xFAD mice did not discriminate between the two objects, spending equal time with both objects (Figure 4C; gray vs. green). Injection of AAV-SecNIa rescued this deficit, restoring recognition preference to levels comparable with WT mice (Figure 4C; gray vs. red). These data indicate SecNIa expression ameliorates impaired recognition memory in 5xFAD mice.

Spatial learning was then evaluated using the Morris water maze test over four consecutive days. Escape latency, defined as the time required to locate the hidden platform, was recorded. WT mice exhibited progressively shorter escape latency across the test days, whereas AAV-GFP-injected 5xFAD mice showed no improvement, consistent with severely impaired spatial learning (Figure 4D; green vs. gray). Notably, AAV-SecNIa injection markedly improved spatial learning of 5xFAD mice, with escape latency nearly indistinguishable from WT mice (Figure 4D; red vs. gray).

To assess spatial memory, a probe test was performed. The hidden platform in the water maze was removed and the time spent in the target quadrant was measured. AAV-GFP-injected 5xFAD mice showed no preference for target quadrant, spending approximately one-quarter of the time in the target quadrant, indicative of impaired spatial memory. In contrast, AAV-SecNIa-injected 5xFAD mice spent significantly more time in the target quadrant, comparable to WT mice, demonstrating preserved or improved spatial memory (Figure 4E).

Collectively, these results show that AAV-SecNIa delivery attenuates recognition memory, spatial learning, and spatial memory deficits in 5xFAD mice.

## 4 Discussion

Our group has previously demonstrated the therapeutic potential of NIa in reducing Aβ burden and rescuing cognitive impairments in models of AD (Han et al., 2010; Kim et al., 2012). Those studies suggested a potential role for cytosolic Aβ in AD pathogenesis. Extending this work, we sought to develop a novel therapeutic strategy by engineering NIa for secretion into extracellular spaces, where Aβ accumulates and aggregates into plaques. The rationale was to enable direct targeting and cleavage of extracellular Aβ species, particularly the monomeric and oligomeric forms. To this end, we designed a secretory version of NIa, SecNIa, by incorporating an Igκ leader sequence for secretion and a modified hFc for enhanced stability. We confirmed *in vitro* that SecNIa retained robust Aβ-cleaving activity (Figure 1). When delivered *in vivo* via AAV, SecNIa was efficiently secreted, reduced Aβ burden, and ameliorated cognitive impairments in 5xFAD mice (Figures 2–4).

Interestingly, SecNIa expression was localized primarily to the CA1/2 subregions of the hippocampus, with comparatively little expression in the CA3 and dentate gyrus (DG) (Figure 2B). This is notable given the critical role of CA1 in spatial memory and object-in-context recognition (Stackman et al., 2016; Ásgeirsdóttir et al., 2020), which correlates with the significant improvements we observed in the novel object recognition and Morris water maze tests. While CA3 and DG are involved in pattern completion and separation, respectively (Greene et al., 2013), the limited SecNIa expression in these regions suggests that the robust cognitive benefits observed here largely stem from SecNIa’s action within CA1/2. In some cases, mild expression of SecNIa was visible in the cortical regions dorsal to the hippocampus following AAV administration (Figure 3C). Despite the detection of SecNIa in the cortex, quantitative analysis of Aβ levels by ELISA showed no significant reduction in Aβ_1–40_ and Aβ_1–42_ in both fractions (data not shown).

Additional studies were performed to analyze potential side effects of SecNIa as well as experiments to gain mechanistic insight into the action of SecNIa. To characterize the neuroinflammatory response, we measured both cytokine levels and glial activation in the hippocampus. Despite the foreign origin of SecNIa, an increase in proinflammatory cytokines (IL-1β and TNF-α) was not observed when compared to VLP-injected controls (Supplementary Figure 2). Interestingly, this stable cytokine profile occurred alongside a significant increase in microglial activation in AAV-SecNIa-injected 5xFAD mice, as revealed by anti-Iba1 IHC (Supplementary Figure 3). To investigate other potential pathways affected by SecNIa, western blotting was performed using antibodies against fragments of APP and autophagy proteins (Supplementary Figures 4–5). Analysis revealed that SecNIa did not modulate autophagy or APP processing, suggesting that the action of SecNIa is independent of these pathways. For a clearer mechanistic picture, future studies are warranted on clearance of the peptides formed by the cleavage of Aβ by SecNIa.

We also compared SecNIa with neprilysin (NEP), a well-characterized endogenous Aβ-degrading protease. AAV-mediated NEP expression in 5xFAD mice produced strong reductions in Aβ plaques (Supplementary Figures 6B–F), consistent with prior studies. However, NEP expression failed to improve cognitive performance: AAV-NEP–treated 5xFAD mice showed only a modest, non-significant improvement in novel object recognition and continued to exhibit severe impairments in the Morris water maze test (Supplementary Figures 7C–E). These results parallel findings by Meilandt et al. (2009), who also reported dissociation between plaque reduction and behavioral rescue in AD model mice treated with NEP. Interestingly, biochemical analysis following behavior tests at 9 months of age, AAV-SecNIa and AAV-NEP-treated 5xFAD mice displayed no difference in total Aβ_1–42_ levels compared to AAV-GFP-treated controls (Supplementary Figure 8), suggesting overwhelming amyloid pathology in 5xFAD (Tg6799) mice at this time point. Nevertheless, SecNIa treatment at 2 months provided a durable cognitive benefit that NEP did not.

Differences in Aβ oligomeric species across AD mouse models may help explain the reported discrepancies regarding the efficacy of NEP. We have previously shown that NIa can cleave oligomeric Aβ, that NEP cannot (Shin et al., 2014). Moreover, studies have suggested that oligomer concentrations and properties vary significantly between AD mouse models (Kass et al., 2022; Purro et al., 2023). Since the oligomeric landscape of 5xFAD mice has not been fully characterized, it is possible that the specific oligomers driving pathology in this model were more susceptible to degradation by SecNIa compared to NEP.

In summary, we successfully engineered SecNIa, a secretory protease that effectively reduces Aβ burden and rescues cognitive deficits in 5xFAD mice. The consistent reductions in both soluble and insoluble Aβ, coupled with significant improvements in recognition and spatial memory, underscore SecNIa’s therapeutic potential. By directly targeting extracellular Aβ species—including oligomers that may evade degradation by NEP—SecNIa offers a complementary and potentially superior approach to existing Aβ-degrading strategies.

### 4.1 Limitations

This study was conducted exclusively in the 5xFAD model which represents aggressive familial AD, and it remains to be determined whether SecNIa would show comparable efficacy in other AD or aged models. Also, 5xFAD female mice display significantly faster and greater accumulation of Aβ (Oakley et al., 2006). Indeed, when female 5xFAD mice at 2 months of age were stereotaxically administered AAV-SecNIa, we could only observe a downward trend in hippocampal Aβ levels 2 months post-injection (data not shown). Due to the sexual dimorphism present in the severity of AD pathology in 5xFAD mice, only male mice were utilized for this study.

Although we observed reductions in both soluble and insoluble Aβ, we did not directly detect different oligomeric species, which could provide additional insight into SecNIa’s effects on the most neurotoxic assemblies. In addition, while we confirmed robust cleavage activity and lack of overt toxicity, the potential off-target proteolysis, immunogenicity, and long-term safety of SecNIa remain to be investigated.

### 4.2 Translational perspective

From a therapeutic standpoint, SecNIa introduces a novel class of protease-based biologics for AD. Compared with antibody-based immunotherapies, which primarily enhance clearance via microglial phagocytosis, SecNIa acts directly and catalytically on extracellular Aβ, potentially offering greater potency at lower doses. Importantly, our data suggest that SecNIa retains specificity without overt toxicity *in vivo*, but further safety profiling—including off-target cleavage, immune activation, and long-term tolerability—will be critical.

AAV-mediated expression provided sustained local delivery in our model, but translation to humans raises additional considerations. While AAV vectors are under clinical evaluation for CNS diseases, invasive stereotactic injection limits scalability. Alternative strategies could include intrathecal or intracisternal administration, or development of a recombinant SecNIa protein stabilized for systemic delivery and engineered to cross the blood–brain barrier. Given recent advances in delivery platforms such as BBB-penetrating nanobodies, exosomes, and circular RNA-based expression systems, SecNIa could be deployed through multiple clinically viable modalities.

Taken together, our findings position SecNIa as a promising candidate for therapeutic development in AD. By directly targeting extracellular Aβ with high specificity, SecNIa provides a mechanistically distinct and potentially more effective strategy to modify disease course compared with existing immunotherapies or endogenous protease augmentation.

## Data Availability

The raw data supporting the conclusions of this article will be made available by the authors, without undue reservation.
