# Supplementary material for: Secretory form of viral protease NIa ameliorates amyloid-β pathology and cognitive deficits in a mouse model of Alzheimer’s disease
Source: Front Aging Neurosci. 2026 Apr 22;18:1720518. doi: 10.3389/fnagi.2026.1720518 (PMC13143923; doi:10.3389/fnagi.2026.1720518)
Supplement: Supplementary file 1 [file Data_Sheet_1.pdf]

Supplementary Data

Secretory form of viral protease NIa ameliorates Aβ pathology and cognitive deficits in a mouse model of Alzheimer's disease

Euy Jun Park, Bo-Ram Mun, Sung Yoon Kim, Muthukumar Elangovan, Sung Bin Kim, Won-Seok Choi, and Woo Jin Park

Supplementary figure 1.

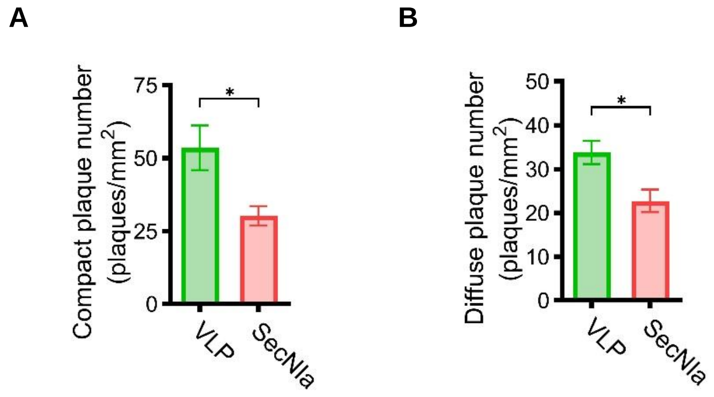

Supplementary figure 1. SecNIa reduces plaques of varying morphology (A) The number of compact plaques and (B) diffuse plaques in the hippocampus of 4-month-old 5xFAD mice brain. Student's *t*-test: * $p < 0.05$. Bars and error bars represent means ± SEM. Number of animals used (all male) 5xFAD/VLP, 4; 5xFAD/SecNIa, 4.

Supplementary figure 2.

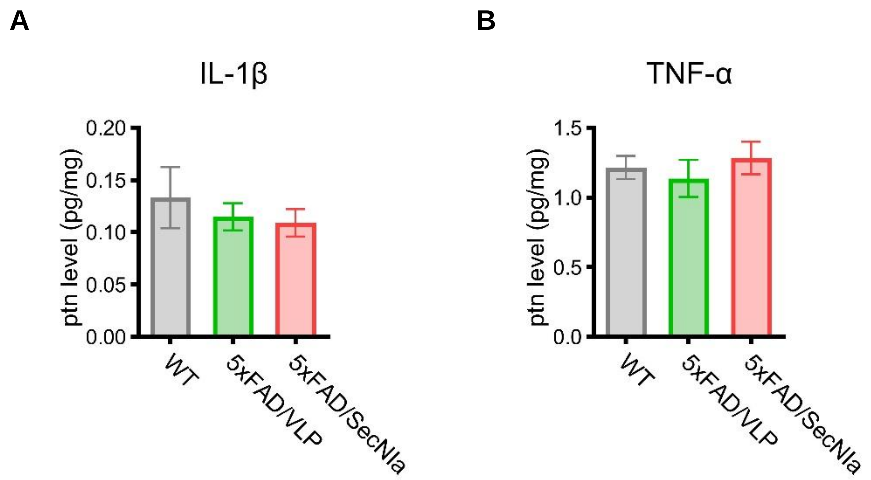

Supplementary figure 2. SecNIa does not induce interferon-mediated inflammatory response (A) Pro-inflammatory cytokine levels of IL-1β and (B) TNF-α in 4-month-old WT and 5xFAD mice hippocampal lysates measured by ELISA. Bars and error bars represent means ± SEM. Number of animals used (all male) WT, 3; 5xFAD/VLP, 4; 5xFAD/SecNIa, 4.

Supplementary figure 3.

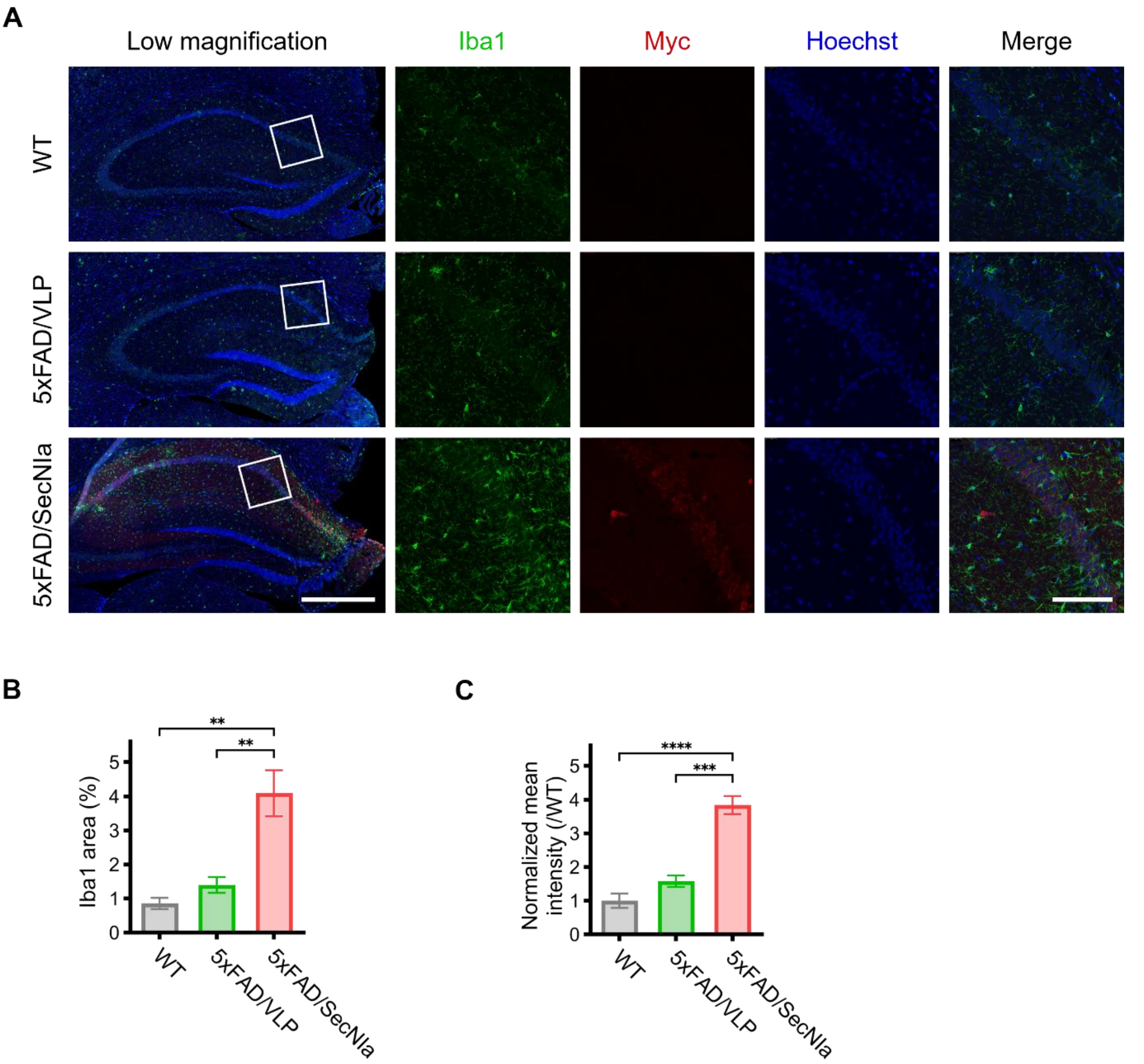

Supplementary figure 3. SecNIa induces significant microglial activation (A) Representative images of 4-month-old WT and 5xFAD coronal sections subjected to IHC. Scale bar, low magnification, 500 μm; high magnification, 100 μm. (B-C) Quantitative analysis of anti-Iba1 IHC images in high magnification: (B) Percentage area covered by Iba1 signal and (C) Mean green fluorescence intensity normalized to WT. One-way ANOVA: ** $p < 0.01$, *** $p < 0.001$, **** $p < 0.0001$. Bars and error bars represent means ± SEM. Number of animals used (all male) WT, 3; 5xFAD/VLP, 4; 5xFAD/SecNIa, 4.

Supplementary figure 4.

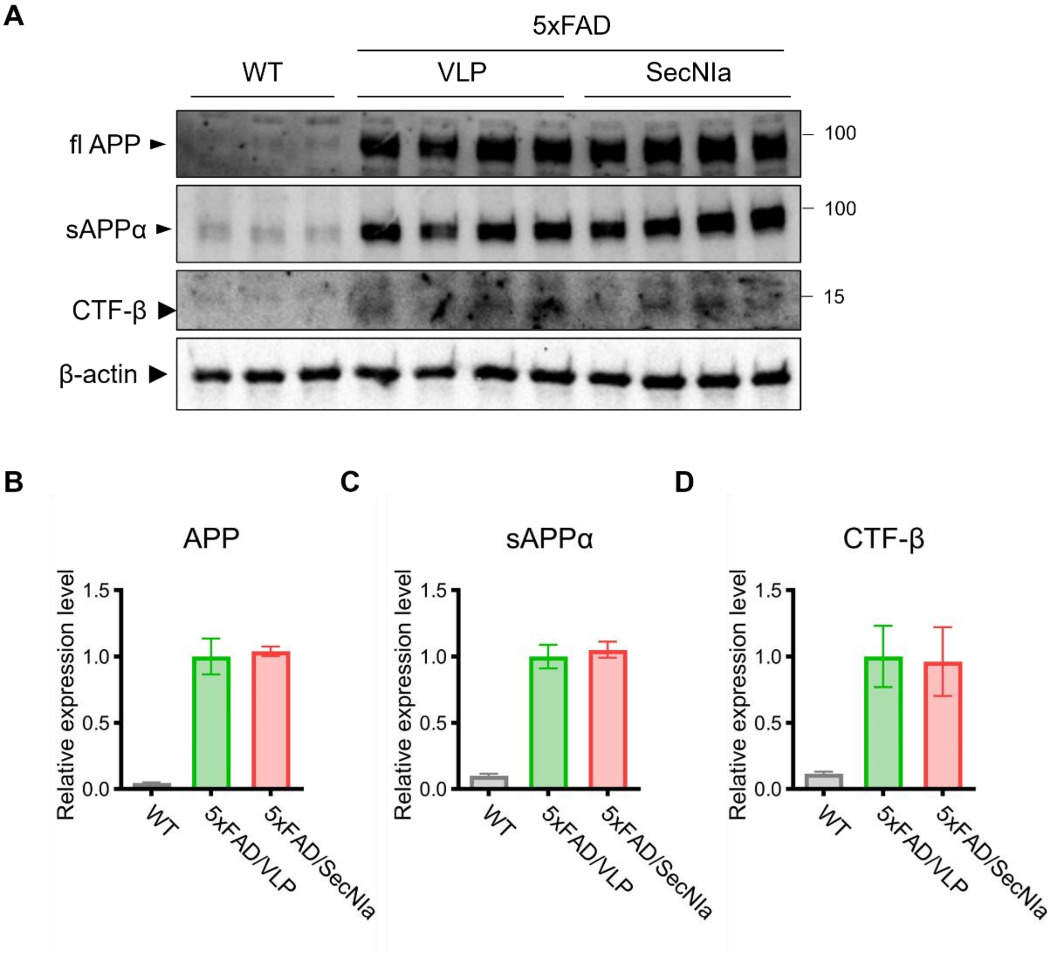

Supplementary figure 4. SecNIa does not affect APP processing (A) Western blotting of hippocampal lysates from 4-month-old WT and 5xFAD mice. Relative expression levels of (B) full length APP, (C) soluble APP α (sAPPα), and (D) C-terminal fragment β of APP (CTF- β). Bars and error bars represent means ± SEM. Number of animals used (all male) WT, 3; 5xFAD/VLP, 4; 5xFAD/SecNIa, 4.

Supplementary figure 5.

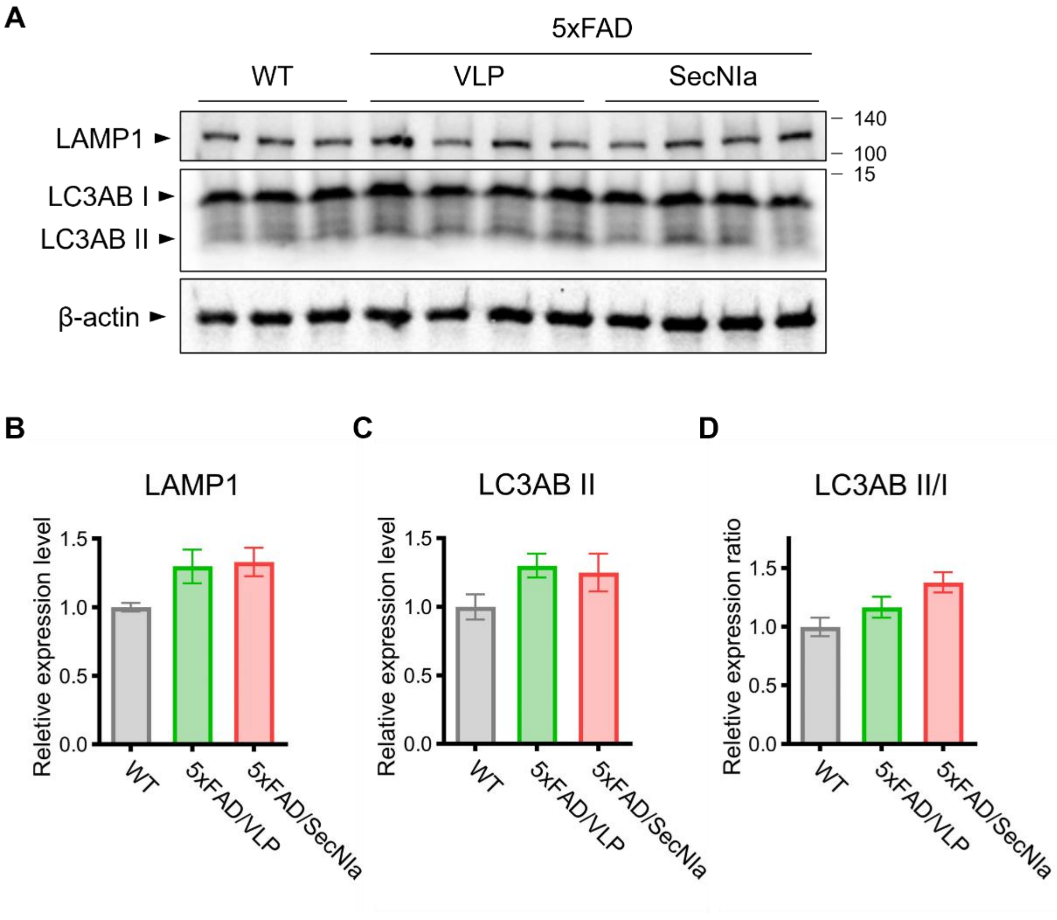

Supplementary figure 5. SecNIa does not affect autophagy (A) Western blotting of hippocampal lysates from 4-month-old WT and 5xFAD mice. Relative expression levels of (B) LAMP1, (C) LC3A/B II, and (D) LC3A/B II/I ratio. Bars and error bars represent means ± SEM. Number of animals used (all male) WT, 3; 5xFAD/VLP, 4; 5xFAD/SecNIa, 4.

Supplementary figure 6.

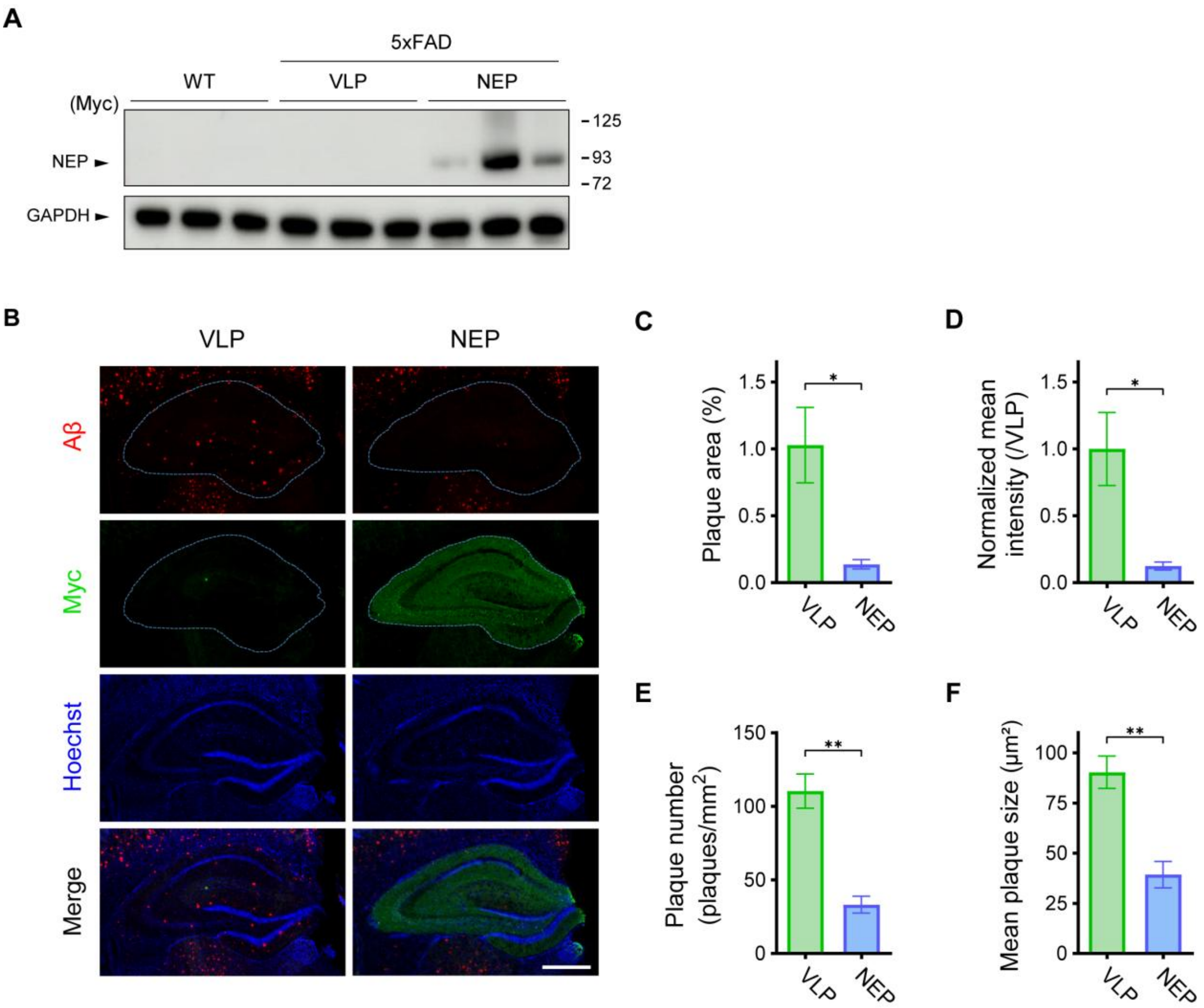

Supplementary figure 6. AAV-NEP prevents plaque formation in 5xFAD mice (A) Western blotting of hippocampal lysates from 4-month-old WT mice and 5xFAD mice injected with AAV-VLP or AAV-NEP under non-reducing conditions. (B) Representative 20 μm coronal mouse brain sections subjected to IHC with anti-Aβ (N-term, red) and anti-Myc-tag (green). Scale bar, 500 μm. (C-F) Quantitative analysis of anti-Aβ IHC images in the hippocampus: (C) Percentage area covered by plaques, (D) Mean red fluorescence intensity normalized to VLP, (E) Plaque count per mm^2 tissue, and (G) Mean plaque size. Student's *t*-test: * $p < 0.05$, ** $p < 0.01$. Bars and error bars represent means ± SEM. Number of animals used (all male) 5xFAD/VLP, 4; 5xFAD/NEP, 4.

Supplementary figure 7.

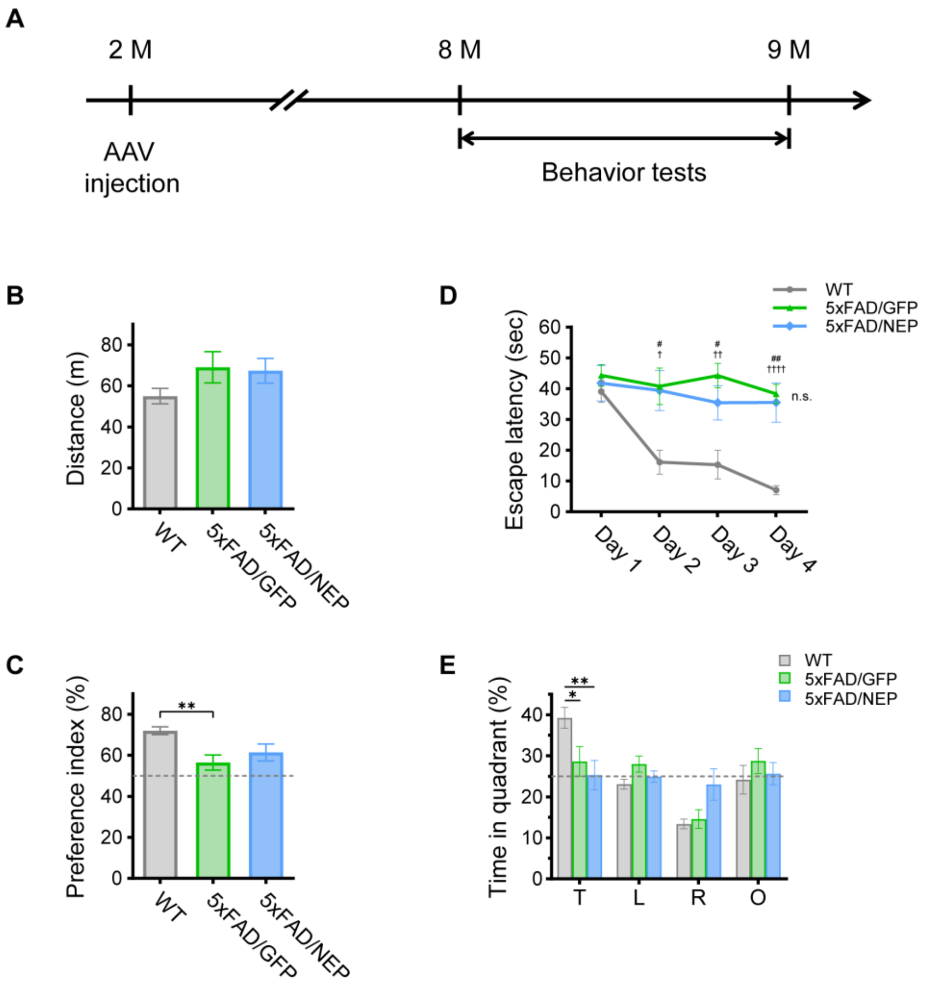

Supplementary figure 7. AAV-NEP confers no cognitive improvements in 5xFAD mice (A) Experimental scheme of stereotaxic AAV delivery and behavior tests. 5xFAD mice were injected at two months of age and subjected to behavior tests six months later. (B) Open-field test assessing locomotive activity. (C) Novel object recognition test evaluating recognition memory. One-way ANOVA: ** $p < 0.01$. (D) Morris water maze tests of spatial learning. Two-way repeated measures ANOVA: $^{\#} p < 0.05$, $^{\#\#} p < 0.01$ WT vs 5xFAD/NEP; $^{\dagger} p < 0.05$, $^{\dagger\dagger} p < 0.01$, $^{\dagger\dagger\dagger\dagger} p < 0.0001$ WT vs 5xFAD/GFP. n.s., no significance. (E) Probe test assessing spatial memory. Two-way ANOVA: * $p < 0.05$, ** $p < 0.01$. Bars or data points represent means ± S EM. Number of animals used (all male) WT, 7; 5xFAD/GFP, 8; 5xFAD/NEP, 7.

Supplementary figure 8.

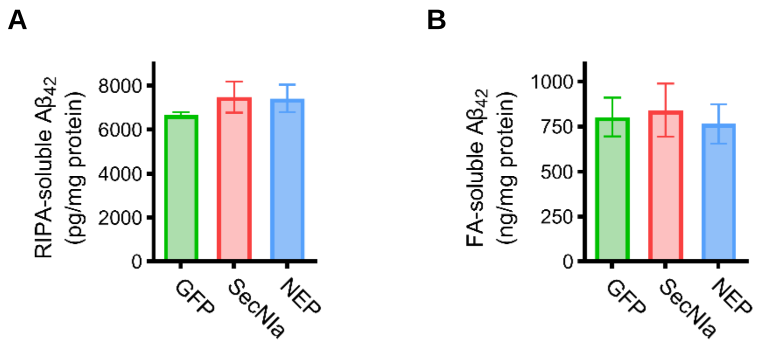

Supplementary figure 8. 5xFAD mice display overwhelming $A\beta_{1\text{-}42}$ levels at 9 months (A-B) 5xFAD mice were injected at two months of age and sacrificed seven months post-injection following behavior studies. (A) RIPA and (B) FA-soluble fractions of hippocampal lysates were subjected to $A\beta_{1\text{-}42}$ ELISA. Bars and error bars represent means ± SEM. Number of animals used (all male) 5xFAD/GFP, 8; 5xFAD/SecNIa, 6; 5xFAD/NEP, 7.
